# Standardization of Germinated Oat Extracts and Their Neuroprotective Effects Against Aβ_1-42_ Induced Cytotoxicity in SH-SY5Y Cells

**DOI:** 10.3390/molecules30153291

**Published:** 2025-08-06

**Authors:** Yu-Young Lee, In-Su Na, Jeong-Eun Kim, Jae-Gwang Song, Chae-Eun Han, Hyung-Wook Kim, Soon-Mi Shim

**Affiliations:** 1 Department of Food Science, National Institute of Crop Science, Rural Development Administration, Suwon 16429, Republic of Korea; leeyy260@korea.kr; 2 Department of Food Science and Biotechnology, Sejong University, Seoul 05006, Republic of Korea; 23110112@sju.ac.kr (I.-S.N.); 24110208@sju.ac.kr (J.-E.K.); 3 Department of Biological Science and Technology, Sejong University, Seoul 05006, Republic of Korea; songjg@sejong.ac.kr (J.-G.S.); ghwnsdlvv@sejong.ac.kr (C.-E.H.); kimhyung@sejong.ac.kr (H.-W.K.)

**Keywords:** germinated oat extracts, avenanthramides, Aβ_1-42_, human neuroblastoma cell, memory improvement

## Abstract

The present study aimed to standardize germinated oat extracts (GOEs) by profiling avenanthramides (AVNs) and phenolic acids and evaluate their neuroprotective effects against Aβ_1-42_-induced cytotoxicity in human neuroblastoma (SH-SY5Y) cells. GOEs were standardized to contain 1652.56 ± 3.37 µg/g dry weight (dw) of total AVNs, including 468.52 ± 17.69 µg/g AVN A, 390.33 ± 10.26 µg/g AVN B, and 641.22 ± 13.89 µg/g AVN C, along with 490.03 ± 7.83 µg/g dw of ferulic acid, using a validated analytical method. Treatment with AVN C and GOEs significantly inhibited Aβ_1-42_-induced cytotoxicity (*p* < 0.05). Furthermore, both AVNs and GOEs markedly reduced Aβ_1-42_-induced reactive oxygen species (ROS) generation in SH-SY5Y cells, showing significant scavenging activity at concentrations of 25 μg/mL (AVNs) and 50 μg/mL (GOEs) (*p* < 0.05). RT-PCR analysis revealed that AVNs and GOEs effectively downregulated the expression of inflammation- and apoptosis-related genes triggered by Aβ_1-42_ exposure. These findings suggest that GOEs rich in AVNs may serve as a potential functional ingredient for enhancing memory function through the inhibition of neuroinflammation and oxidative stress.

## 1. Introduction

Alzheimer’s disease is a progressive neurodegenerative disorder characterized by memory loss and cognitive decline, primarily associated with the formation of senile plaques, neurofibrillary tangles, and the accumulation of β-amyloid (Aβ) fibrils (Uwishema, Mahmoud [1]. The percentage of people with Alzheimer’s disease increases with age, and the annual number of new cases of Alzheimer’s and other forms of dementia is expected to double by 2050 [2]. Several hypotheses have been proposed to explain its pathogenesis, including the cholinergic hypothesis, which attributes cognitive decline to the loss of cholinergic neurons and reduced acetylcholine levels; the tau hypothesis, which involves the aggregation of hyperphosphorylated tau proteins into neurofibrillary tangles; and the neuroinflammatory hypothesis, which emphasizes chronic neuroinflammation driven by activated microglia and astrocytes [3,4,5]. It is well-known that the primary trigger for the growth of Alzheimer’s disease is the deviant metabolism of the amyloid precursor glycoprotein and the subsequent accumulation of soluble oligomers Aβ_1-40_ and Aβ_1-42_ [6,7]. It was also confirmed that Aβ_1-42_ correlated with the formation of a neuroinflammatory mediator, such as reactive oxygen species (ROS) and the tumor necrosis factor (TNF)-α, which can cause neuronal cell apoptosis [8]. Many studies suggest, for this reason, that the prevention of oxidation and activation of anti-inflammation could be critical factors in order to improve Alzheimer’s disease [9,10]. For instance, flavonoids, such as quercetin, which are well-known antioxidants, provide neuroprotective effects by inhibiting the apoptotic cell death of the SH-SY5Y cell against H_2_O_2-_induced oxidative stress [11,12]. The study also found that oral intake of quercetin before intracerebroventricular (i.c.v.) injection of aggregated Aβ_25-35_ improved memory function in mice via a behavioral test.

Oats (*Avena sativa* L.) are the most cultivated grains after corn, beans, rice, wheat, and barley, and they are a sufficient source of nutrients, which include soluble fibers, beta-glucan, vitamin B, unsaturated fatty acids, proteins, and minerals [13,14]. Oats also contain various types of polyphenols, such as *p*-coumaric acid, ferulic acid, and caffeic acid, which are considered to be antioxidants [15]. Avenanthramides (AVNs), which are a special group of phenolic alkaloids, are in particular commonly found in oats [16]. AVNs themselves are known as antipathogens (phytoalexin), which are created in response to exposure to pathogens, due to their strong antioxidant and anti-inflammatory activity [17,18]. There are twenty different forms of AVNs that are identified in oat extracts, and three types are major forms, which consist of a 5-hydroxyanthranilic acid and a cinnamic acid, such as *p*-coumaric acid (AVN A), ferulic acid (AVN B), and caffeic acid (AVN C) [18,19]. There is a considerable evidence to suggest that oats protect neuronal cells against inflammation as well as improve memory loss due to dementia [20,21,22]. It was found that oat extract enriched AVNs increased the reduced form of glutathione in plasma by antioxidant activity, which resulted in the improvement of CNS differentiation and proliferation [18,23]. The inhibitory effects of AVN-C on ROS generation were observed in PC12 cells exposed to H_2_O_2_ and 6-hydroxydopamine hydrobromide, where AVN-C attenuated ROS production in a dose-dependent manner. These cells, derived from adrenal pheochromocytoma, showed a significant reduction in oxidative stress upon AVN-C treatment [24]. A previous study demonstrated the inhibitory effects on Aβ deposition by treating H2-22 cells and the brains of Tg-5XFAD Alzheimer’s disease mice with AVN A and oat seed extract [25]. Furthermore, it was also reported that the content and bioavailability of AVNs increased with germinating cereals [26,27]. In this context, “germinating cereals” refer to grains that have undergone controlled soaking and incubation to induce sprouting, whereas “non-germinating cereals” indicate grains that have not been subjected to such treatment [28]. The oral intake of oat extracts in particular promoted the peak plasma concentration of AVNs after 2 h, which indicated the highest bioavailability [18,29]. However, an accurate mechanism in regard to protecting the Aβ-impaired synaptic function by AVN-enriched germinated oat extracts (GOEs), which shows promise as a functional ingredient, is not fully understood. Thus, the current study firstly standardized the GOEs as a functional ingredient by profiling the AVNs and phenolic acids using the validated analytical method with high-performance liquid chromatography (HPLC) with an ultra-violet (UV) detector.

The human neuroblastoma SH-SY5Y cell line is a commonly used model in regard to screening neuroprotective therapeutic agents [17,30]. We investigated the AVN and GOEs in order to determine their efficacy in alleviating apoptosis and the ROS production against Aβ-induced SH-SY5Y cell damage. In addition, we measured the expression of the nuclear factor kappa-light-chain-enhancer of activated B cells (NF-κB), the anti-apoptotic enzyme Bcl-2, and the pro-apoptotic enzyme Bax in SH-SY5Y treated with Aβ_1-42_ in order to examine the effects of the GOEs treatment on inflammation and apoptosis.

## 2. Materials and Methods

### 2.1. Chemicals and Reagents

The analytical standards of avenanthramide (AVN) A, B, and C were purchased from Sigma-Aldrich (≥98%, St. Louis, MO, USA). The analytical grades of vanillic acid, caffeic acid, ferulic acid, and *p*-coumaric acid were obtained from Sigma-Aldrich (≥98%, MO, USA). The formic acid (HCOOH) was purchased from Fisher Scientific (Morristown, NJ, USA), and the acetonitrile (ACN), methanol, and water for HPLC grade were obtained from J.T. Baker (Phillipsburg, NJ, USA). The distilled water was obtained from Joylife (Busan, Republic of Korea), and the ethanol was purchased from Daehan Ethanol Life (Hwaseong, Republic of Korea). All the chemicals were liquid chromatography (LC) grade. The minimum essential medium (MEM) and the fetal bovine serum (FBS) were purchased from HyClone™ (Marlborough, MA, USA). The phosphate-buffered saline (PBS) was obtained from Biowest (Nuaille, France), and the penicillin-streptomycin was obtained from Corning Inc. (Corning, NY, USA). The 3-[4,5-dimethylthiazol-2yl]-2,5-diphenyl-tetrazolium bromide and the 2′,7′-Dichlorodihydrofluorescein diacetate (DCFH-DA) were purchased from Sigma-Aldrich (MO, USA). The human β-amyloid (Aβ_1-42_) peptide was obtained from Abcam (Cambridge, UK). TRIzol reagent was obtained from Invtrogen (Waltham, MA, USA), the isopropanol was acquired from Duksan (Seoul, Republic of Korea), and the nuclease-free water (AM9937) was purchased from Bio Basic Inc. (Markham, ON, Canada).

### 2.2. Sample Preparation for Germinated Oat Extracts (GOEs)

The *Daeyang* germinated oats were harvested from Gyeongju-gun, South Korea, which is Region 1, during the spring of 2021, and they were kindly provided by Doobo Food Company (Seoul, Republic of Korea). The avenanthramides from *Daeyang* germinated oats were extracted, which is described in a previous study, with slight modifications [31]. The *Daeyang* germinated oats were ground using a food grinder (Hanil, Bucheon, Republic of Korea), and an aliquot amount of the ground sample (100 g) was mixed with 30% ethanol to 10 times the ratio of the sample weight, which was then extracted at 40 °C for 6 h. The extracts were filtered using 8 µm of Whatman No. 2 filter paper (Whatman International Ltd., Maidstone, UK). They were then concentrated using a vacuum rotary evaporator (Sunileyela, Seongnam, Republic of Korea) until the ethanol solvent was fully removed by evaporation. The concentrated samples were sterilized using autoclaves at 90 °C for 30 min, which was followed by spray drying at 140 °C of the inlet and 45 °C of the outlet temperature using a two-fluid nozzle spray-dryer (SD1000, Eyela, Tokyo, Japan). The dried powder was then stored at 4 °C for further use.

### 2.3. Qualitative and Quantitative Analysis of Avenanthramides (AVNs) in Daeyang Germinated Oat Extracts (GOEs) by HPLC-UV

Identification of the avenanthramides in the GOEs was performed using High-Performance Liquid Chromatography-Ultraviolet (HPLC-UV) (Ultimate 3000, Thermofisher, Waltham, MA, USA), which was slightly modified from a previous method [31]. The systems were utilized with a reverse phase column of YMC-PACK ODS-A (150 × 4 mm, 5.0 μm, YMC, Kyoto, Japan), which was maintained at 35 °C. An aliquot amount of the dried sample (10 mg) was dissolved in 1 mL of 50% MeOH, which was diluted using 100% acetonitrile (ACN) to a 10:1 (*v*/*v*%) ratio for the HPLC-UV analysis that was followed by filtering with 0.45 μm polytetrafluoroethylene (PTFE) syringe filters. The mobile phase consisted of solvent A, which was water that contained 5% ACN with 0.1% formic acid, and solvent B, which was ACN with 0.1% formic acid, and they were eluted at a flow rate of 1 mL/min. The mobile phase gradient began with 100% solvent A, which followed the following order: 0–32.5 min with 100–87% of solvent A, 32.5–69 min with 87–70% of solvent A, 69–75 min with 70–90% of solvent A, and 75–80 min with 90–100% of solvent A, and it was then equilibrated back with 100% of solvent A within 5 min. All the analyses were conducted with a 10 μL injection and were monitored at a wavelength of 330 nm. AVN A, B, and C were quantified by comparisons of their peaks with the external standard curves of those analytical standards.

All analyses were conducted with a 10 µL injection volume. Each sample was analyzed in triplicate, and results are expressed as mean ± standard deviation (SD) from three independent experiments.

#### Analytical Method Validation

The HPLC-UV analytical method of AVN A, B, and C was validated for linearity, accuracy, precision, and sensitivity, which included the limit of detection (LOD) and the limit of quantification (LOQ). The linearity was identified from three replicate analyses at five concentrations, which included 1, 5, 10, 25, and 50 μg/mL, of AVN A, B, and C. The correlation coefficient (*r*^2^) was calculated using the regression equation in Microsoft Excel 365. The accuracy was determined by the degree of agreement between a measured concentration and a nominal concentration. In total, 10 to 100 µg/mL of AVN A, B, and C were analyzed for five replicates, which were calculated using the formula that is provided below.

(1) $$\%\mathrm{Error}=\frac{\mathrm{Measured}\;\mathrm{concentration}-\mathrm{Nominal}\;\mathrm{concentration}}{\mathrm{Nominal}\;\mathrm{concentration}}\times 100$$

(2) $$Accuracy\;\left(\%,\mathrm{recovery}\;\mathrm{rate}\right)=\left(100-Error\right)$$

The precision was expressed by repeatability, which was the approximation of the results that were obtained by the same operator, measuring system, condition, and laboratory over a brief period. The repeatability was conducted by using 10 to 100 µg/mL of AVN A, B, and C, which was replicated 5 times. The repeatability of the analytical method was calculated by the coefficient of variation (%CV), which is the same as the percent of relative standard deviation (%RSD).

(3) $$\%CV=\frac{S.D.\;\mathrm{of}\;\mathrm{measured}\;\mathrm{concentration}}{\mathrm{Mean}\;\mathrm{of}\;\mathrm{measured}\;\mathrm{concentration}}\times 100$$

S.D. is the standard deviation.

The sensitivity was evaluated by the limit of detection and quantification (LOD and LOQ) by the calibration curve.

(4) $$LOD\;\left(\mathrm{Limit}\;\mathrm{of}\;\mathrm{dectection}\right)=3.3\times \frac{S_{y}}{S}$$

(5) $$LOQ\;\left(\mathrm{Limit}\;\mathrm{of}\;\mathrm{quantification}\right)=10\times \frac{S_{y}}{S}$$

S_y_ is the standard deviation of the y-intercepts of the regression curves, and S is the slope of the calibration curve.

### 2.4. Identification of Phenolic Acids in Daeyang Germinated Oat Extracts (GOEs)

An optimized HPLC analysis system was modified from a previous method for the identification of phenolic acids in the GOEs [15]. The samples were analyzed in an Ultimate 3000 HPLC system (Thermofisher, Waltham, MA, USA) on a reversed-phase column (YMC-PACK ODS-A, 150 × 4.6 mm, 5.0 μm, YMC, Kyoto, Japan). The solvent system consisted of 1% acetic acid in water (A) and 1% acetic acid in 100% methanol. The column oven was maintained at 30 °C, and the flow rate was 0.9 mL/min. The injection volume was 10 µL, and the linear gradient conditions were as follows: 0–1.5 min with 100–100% of solvent A, 1.5–7 min with 100–80% of solvent A, 7–13 min with 80–80% of solvent A, 12–44.5 min with 80–50% of solvent A, 44.5–47 min with 50–100% of solvent A, and 47–50 min with 100–100% of solvent A. The initial condition of the gradient was adjusted for 3 min by equilibration.

### 2.5. Human Neuroblastoma Cell (SH-SY5Y) Culture

The human neuroblastoma cell line, SH-SY5Y, was purchased from the Korean Cell Line Bank (KCLB, Seoul, Republic of Korea). The cells were seeded into 100 pi culture plates at a density of approximately 1–2 × 10^6^ cells per plate, allowing them to reach 70–80% confluency within 2–3 days. Cells were cultivated in minimum essential medium (MEM) containing 10% fetal bovine serum (FBS) and 1% penicillin streptomycin (P/S) in a humidified atmosphere of 5% CO_2_ in 95% air at 37 °C. The medium was refreshed every other day, and it was sub-cultured when it reached 70 to 80% confluency. All the samples were treated at a confluence of up to 90%.

### 2.6. Preparation and Aggregation of Aβ_1-42_ Peptide

The aggregation of Aβ_1-42_ peptide was performed according to a previous method [7]. The lyophilized human Aβ_1-42_ peptide was dissolved to 200 µM with a 1% ammonia solution. The peptide was diluted with PBS to a concentration of 1 mg/mL for a longer preservation of Aβ_1-42_. The stock solution was kept at −80 °C for further use. The aggregated Aβ_1-42_ was diluted with a cell culture medium for treatments.

### 2.7. Cell Viability Using an MTT Assay

The MTT assay was performed using the specifications from a previous study with slight modifications [32]. Cell viability was assessed using the MTT assay to evaluate the protective effects of AVNs (A, B, and C) and GOEs against Aβ_1-42_-induced cytotoxicity [33]. The 1 × 10^5^ cells were seeded in each well of the 96-well cell culture plate with a cell culture medium with 10% fetal bovine serum (FBS) and 1% penicillin-streptomycin, and they were incubated for 24 h at 5% CO_2_ and 37 °C. After that, a 1 to 25 µg/mL AVNs standard and 1 to 50 µg/mL GOEs were diluted in the culture medium (MEM), and they were treated with the control group, which included only MEM for 24 h. Aβ_1-42_ (20 µM) was treated in order to identify the protective effects of the AVNs and the GOEs against the β-amyloid-induced apoptosis. The treatment was removed after a 24 h incubation, and a 10-fold diluted MTT solution (5 mg/mL) was added for 2 h. The MTT solution was removed after confirming the formation of the formazan, and it was diluted with 200 µL of DMSO. The cell viability was obtained by using a microplate (Varioskan Flash, Thermo Scientific, San Jose, CA, USA) at 570 nm, which was calculated using the equation that is provided below.

(6) $$\mathrm{Cell}\;\mathrm{viability}\;\left(\%\right)=\frac{\mathrm{Absorbance}\;\mathrm{of}\;\mathrm{treatment}}{\mathrm{Absorbance}\;\mathrm{of}\;\mathrm{control}}\times 100$$

### 2.8. Measurement of Reactive Oxygen Species (ROS) Induced by Aβ_1-42_

The intracellular ROS levels were measured using a method from a previous study with slight modifications [33]. The 2′7′-DCFH-DA assay was conducted in order to identify the ROS levels that were induced by Aβ_1-42_, and it was reduced by the AVNs, the phenolic acids standard, and the GOEs. The SH-SY5Y cells were seeded in 96-well black cell culture plates at a density of 1 × 10^5^ cells per well for 24 h. Each well was washed using DPBS, and the cells were then treated using 1 to 25 µg/mL AVNs, the phenolic acids standard, and 1 to 50 µg/mL of the GOEs for 24 h in incubation. Aβ_1-42_ (20 µM) was treated for 24 h in order to identify the protective effects of the AVNs and phenolic acids standard and the GOEs against β-amyloid-induced oxidative stress. A 200 µL 2′7′-DCFH-DA solution was added to each well after 24 h of incubation, and it was incubated for 30 min at 5% CO_2_ and 37 °C. After that, each well was washed 2 times with PBS. The intracellular ROS levels were measured using a microplate at 488 nm for excitation and 525 nm for emission, which was calculated using the equation that is provided below.

(7) $$ROS\;\left(\%\right)=\frac{\mathrm{Average}\;\mathrm{of}\;\mathrm{treatment}}{\mathrm{Average}\;\mathrm{of}\;\mathrm{control}}\times 100$$

The DCFH-DA assay reflects the total intracellular oxidative stress rather than distinguishing individual ROS species, as ROS are short-lived and indirectly measured through the formation of the stable fluorescent compound DCF [34].

### 2.9. Sample Preparation for Reverse Transcription Polymerase Chain Reaction

Any pre-existing aggregates were removed by dissolving the Aβ_1-42_ into 5 mM of hexafluoro-2-propanol (HFIP) in order to prepare the oligomeric Aβ_1-42_, which was aliquoted into microcentrifuge tubes. It was then allowed to evaporate in a fume hood for 16 h and stored at −20 °C. Aβ_1-42_ was diluted to 100 µM in a serum-free and phenol-free cell culture medium for the oligomeric samples, and it was incubated at 4 °C for 24 h. The 3.25 × 10^5^ cells were seeded in 6-well cell culture plates and incubated with the cell culture medium for 24 h at 5% CO_2_ and 37 °C. The medium was then removed, and the cells were pre-treated by 1 and 50 µg/mL GOEs and 25 µg/mL AVNs for 24 h. The oligomeric Aβ_1-42_ was treated with 1 µM for 24 h in incubation in order to identify the protective effects of the AVNs and GOEs against oligomeric Aβ_1-42_-induced neuroinflammation and the disruption of the neurotransmitter.

### 2.10. RNA Isolation and Reverse Transcription Polymerase Chain Reaction (RT-PCR) Analysis

The total RNA in the SH-SY5Y cells was extracted using TRIzol (Invitrogen, Carlsbad, CA, USA). The RNA concentration was analyzed using a NanoDrop 2000 spectrophotometer (Thermo Fisher Scientific, Waltham, MA, USA). A total of 500 ng of the SH-SY5Y RNA was reverse-transcribed with High-Capacity RNA to the cDNA Kit (Applied Biosystems, Foster City, CA, USA) by following the manufacturer’s instructions. The RT-PCR was performed using a PowerUp™ SYBR™ Green Master Mix (Applied Biosystems, CA, USA) and a QuantStudio™ 1 Real-Time PCR instrument (Applied Biosystems, CA, USA). The relative expression levels were calculated by using the 2^−ΔΔCT^ method. GAPDH was used as the endogenous control. The primer sequences are shown in Table 1.

### 2.11. Statistical Analysis

All the data was expressed as mean ± SD in triplicate. A one-way analysis of variance (ANOVA) was applied in order to measure the significant differences among the samples at *p* < 0.05 using GraphPad Prism 6.01 software (GraphPad, La Jolla, CA, USA). A *p*-value below 0.05 was considered statistically significant, and the post hoc Tukey test was conducted at the 95% confidence level. Data were analyzed using one-way or two-way analysis of variance (ANOVA), as appropriate, followed by Tukey’s honestly significant difference (HSD) post hoc test for pairwise comparisons. Statistical significance was set at *p* < 0.05.

## 3. Results and Discussion

### 3.1. Bioanalytical Methods Validation of AVN A, B, and C by HPLC-UV

**Figure 1:**
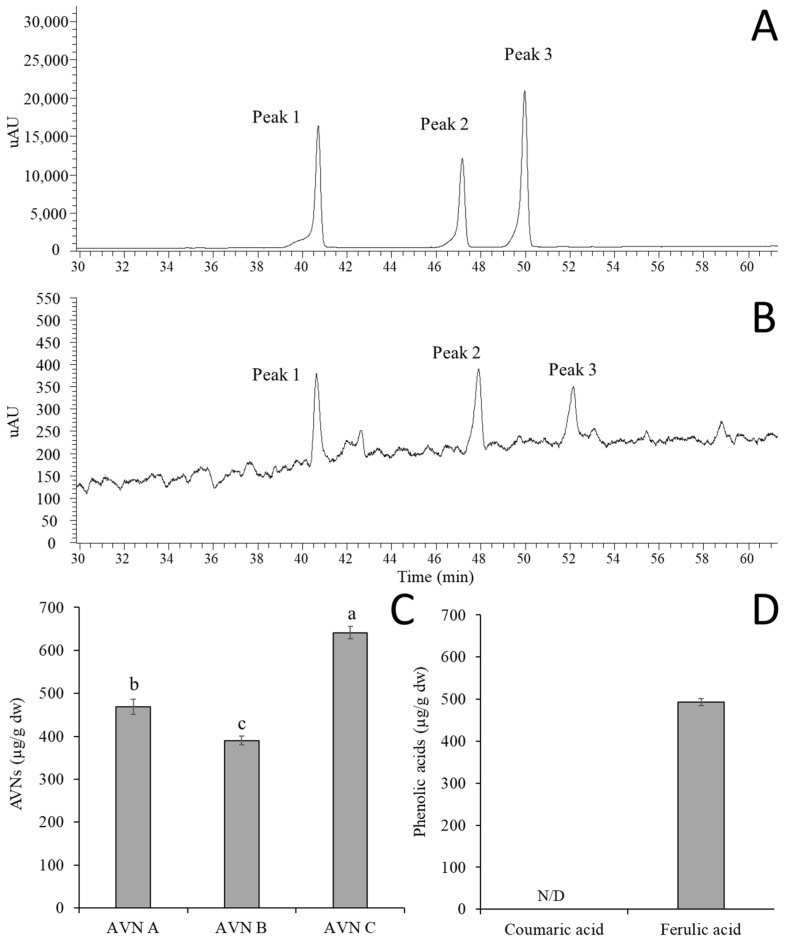
UV chromatograms of AVNs in the standard solution (**A**) and oat extracts (**B**), along with the quantification of AVNs (**C**) and phenolic acids (**D**) from the oat extracts, where peaks 1, 2, and 3 correspond to AVN C, A, and B, respectively. Data are presented as mean ± standard deviation (SD) of [n = 3] independent experiments. Statistical significance among treatment groups was determined using one-way ANOVA (or two-way ANOVA where applicable), followed by Tukey’s honestly significant difference (HSD) post hoc test. Different letters (a, b, c) above the bars indicate statistically significant differences among groups (*p* < 0.05). N/D means not detected, which is below the limit of detection.

The method validation was conducted in order to set the optimal analysis using 1 to 100 µg/mL of the AVNs standard solution. AVN A, B, and C were well separated, which were eluted at 47.16 min, 49.95 min, and 40.29 min, respectively. This indicated that the analytical condition provided specificity, which is shown in Figure 1C. The correlation coefficient (*r*^2^) for each standard solution with the range of 1 to 50 µg/mL was over 0.999. This means the regression equation performed well, as shown in Table 2. Both accuracy and precision of the analytical method were evaluated using 10, 50, and 100 µg/mL standard solutions via repeated measurements for five replicates during a single day, as shown in Table 2. The accuracy was between 98.82~100.47, 100.17~102.70, and 99.70~101.59 for AVN A, B, and C, respectively. This indicates that it is less than a 10% error, which is an accepted reference value. The precision ranged from 1.40 to 4.85% CV, which was estimated by the ratio of the mean and standard deviation for all standard solutions. Table 2 shows that the LOD and LOQ, which were determined in the standard solution, ranged between 0.610 and 0.733 µg/mL for the LOD and 1.848 and 2.223 µg/mL for the LOQ. These results confirmed that the developed analytical method for AVN A, B, and C provided acceptable specificity, linearity, accuracy, precision, and sensitivity.

### 3.2. Standardization of GOEs by Quantifying AVN and Phenolic Acids

It is well documented that avenanthramides function as phytoalexins in oats and are induced by fungal infection, particularly *Puccinia coronata*, as well as by chemical elicitors [35]. The AVNs and phenolic acids from the GOEs, which were followed by extraction, concentration, sterilization, and spray drying, were identified by using the validated analytical method that is described in Table 2. The contents of AVN A, B, and C from the GOEs were 468.52 ± 17.69, 390.33 ± 10.26, and 641.22 ± 13.89 µg/g dw after the spray drying, which are shown in Figure 1C (*p* < 0.05). In addition, the targeted phenolic acids, which included the ferulic acid and *p*-coumaric acid, were identified and quantified from the oat extracts. The *p*-coumaric acid was not detected from the GOEs, but it had 490.03 ± 7.83 µg/g dw of ferulic acid, which is displayed in Figure 1D. The yield (%) of the oat extracts, which was determined by the ratio of the weight of dried powder to the initial weight of germinated oats, was 6.81% at the GOEs.

AVN A, B, and C are widely known as the major components in oats [31]. A previous study found that the AVNs accumulation in oat groat was mostly influenced by the growing location (68 to 77%), which was compared with six Canadian oat cultivars in different locations, and it was the highest when it was grown in crown rust-infected locations [36]. The study also suggested that high temperatures and precipitation tend to exhibit the improved growth of fungal pathogens, which are known to elicit AVNs biosynthesis. The total amount of AVNs in the germinated oat extracts was found to be significantly increased. It was also found that the contents of the AVNs remarkably increased over a 48 h germination process, which is similar to our findings, and it increased AVN C in non-germinated oats from 84.3 ± 3.1 µg/g to 216.0 ± 1.9 µg/g dw by germinating after extracting it with 80% ethanol [37]. A previous study reported that ferulic acid was significantly reduced by the germination process, which is in contrast to the AVNs [38].

AVN C restored damaged memory by relieving memory impairment and reinforcing synapses [39]. The findings from the current study, where germination increased the AVNs and certain types of phenolics in oats, imply that germinated oat extracts (GOEs) could be potent in regards to improving brain function. Thus, we conducted a further study with standardized germinated oat extract (GOE)-enriched AVNs in order to investigate the effects of oat extracts in regards to improving memory function.

### 3.3. Effects of AVNs and GOEs on Aβ_1-42_-Induced Cytotoxicity of SH-SY5Y Cells

**Figure 2:**
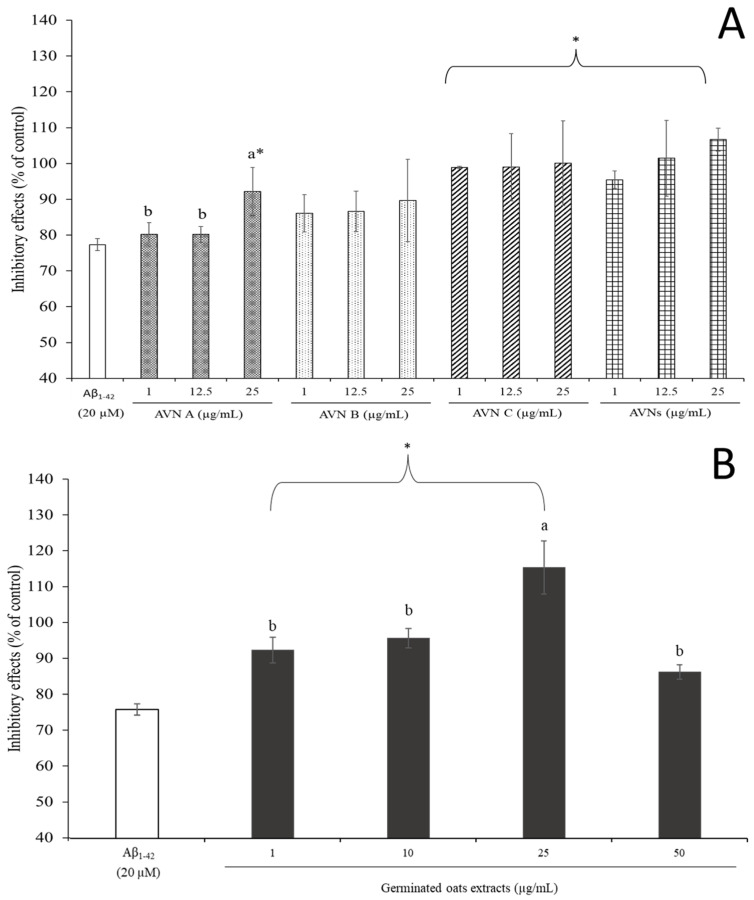
Inhibitory effects (% of control) of the AVNs (**A**) and oat extracts (GOEs) and (**B**) against Aβ_1-42_-induced cytotoxicity in the SH-SY5Y cell. The different letters (a, b) indicate significant differences among concentrations (*p* < 0.05). An asterisk (*) indicates significant differences between the Aβ_1-42_ and each treatment group (*p* < 0.05). Significant difference (HSD) post hoc test.

Aβ_1-42_ ranged from 100 nM to 1 µM in a preliminary study, and it induced a significant decrease in cell viability, which showed 95.15 ± 3.87 to 64.96 ± 1.68% of the control in a dose-dependent manner. An Aβ_1-42_ concentration of 20 µM showed the most toxicity, which was based on this data, and it was chosen for further study. Figure 2 shows the inhibitory effects of various concentrations of AVNs, which were from 1 to 25 µg/mL, and GOEs, which were from 1 to 50 µg/mL, against the Aβ_1-42_-induced cytotoxicity of SH-SY5Y cells. The cell viability was significantly declined to 80.18 ± 3.30% at 1 µg/mL AVN A, 80.16 ± 2.18% at 12.5 µg/mL AVN A, and 92.15 ± 6.72% at 25 µg/mL AVN A (*p* < 0.05), which was 86.07 ± 5.23%, 86.64 ± 5.56%, and 89.66 ± 11.52% at 1, 12.5 and 25 µg/mL AVN B, respectively (*p* < 0.05). The cell viability was 98.90 ± 0.32%, 98.95 ± 9.40%, and 100.04 ± 11.83% at 1, 12.5 and 25 µg/mL, respectively, in regards to AVN C (*p* < 0.05). Furthermore, the cell viability increased 95.45 ± 2.46% at 1 µg/mL AVNs, 101.45 ± 10.55% at 12.5 µg/mL AVNs, and 106.66 ± 3.22% at 25 µg/mL total AVNs (*p* < 0.05), as displayed in Figure 2A. The overall co-treatment of the GOEs increased the cell viability against the cytotoxicity with values of 94.39 ± 3.67%, 97.81 ± 2.75%, 118.30 ± 7.66%, and 88.07 ± 2.01% at 1, 10, 25, and 50 µg/mL GOEs, respectively (*p* < 0.05), as shown in Figure 2B. The inhibitory effects of the AVNs against the Aβ_1-42_-induced cytotoxicity were shown in a dose-dependent manner at AVN A, and it was significantly effective (*p* < 0.05). The co-treatment of AVN C, the total AVNs, and the GOEs, which included 1, 10, and 25 µg/mL, with Aβ_1-42_ was significantly more protective than AVN A and AVB B (*p* < 0.05).

A previous study observed that the AVNs provided a protective effect on the PC12 cell that induced H_2_O_2_ damage. In particular, 10 to 40 µM AVN C displayed a strong protective effect [24]. A similar finding showed the cytotoxicity of Aβ_1-42_ on the SH-SY5Y cells highly decreased at AVN C even at a low dose. The results from the current study imply that the low doses of AVNs and GOEs could mitigate the apoptosis of SH-SY5Y cells induced by Aβ_1-42_.

### 3.4. Effects of SH-SY5Y Cell by AVNs and GOEs from Aβ_1-42_-Induced Oxidative Stress

**Figure 3:**
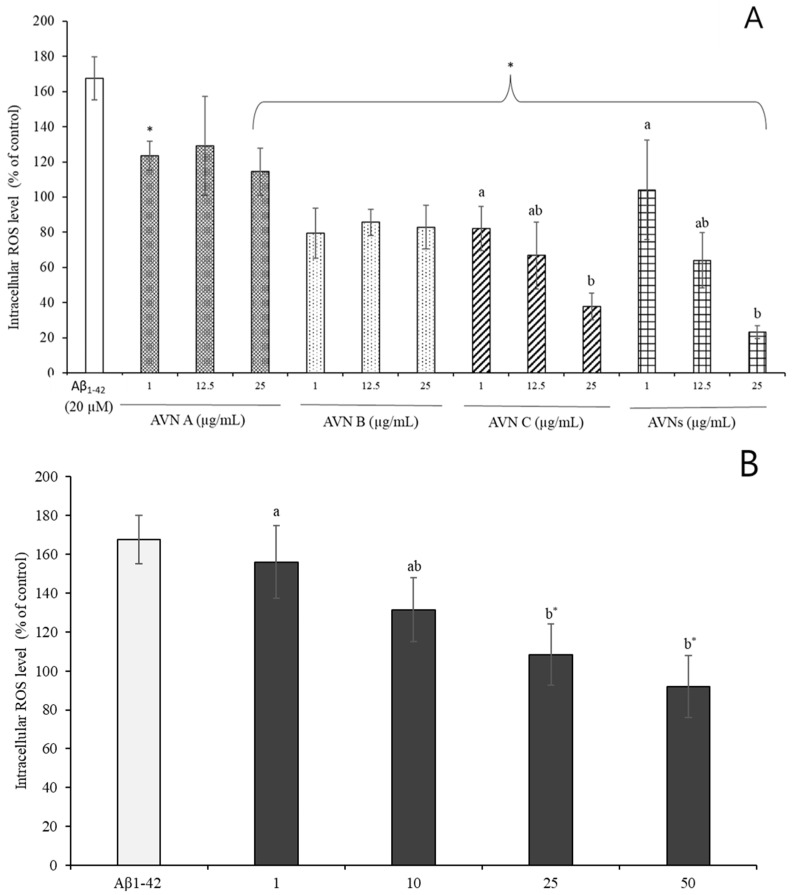
Inhibitory effect of AVNs (**A**) and oat extracts (GOEs) (**B**) on intracellular ROS production induced by Aβ_1-42_. The different letters (a, b) indicate significant differences among concentrations (*p* < 0.05). An asterisk (*) indicates significant differences between the Aβ_1-42_ and each treatment group (*p* < 0.05). Data are presented as mean ± standard deviation (SD) of [n = 3] independent experiments. Statistical significance among treatment groups was determined using one-way ANOVA (or two-way ANOVA where applicable), followed by Tukey’s honestly significant difference (HSD) post hoc test.

The intracellular ROS generation was quantified using DCFH-DA in order to investigate whether the AVNs and GOEs are accountable for the neuroprotection against the Aβ_1-42_-induced generation of reactive oxygen species (ROS). The cellular ROS levels were significantly elevated to approximately 167.50 ± 12.39% compared to the control by the addition of 20 µM Aβ_1-42_ into the SH-SY5Y cells, which is displayed in Figure 3A. The generated ROS by Aβ_1-42_ was overall effectively eliminated, which is shown in Figure 3A, when the AVNs that ranged from 1 to 25 µg/mL were co-treated with Aβ_1-42_. The intracellular ROS level was diminished to 114.46 ± 13.44% at 25 µg/mL AVN A, 82.93 ± 12.30% at 25 µg/mL AVN B, 37.91 ± 7.59% at 25 µg/mL AVN C, and 23.21 ± 3.64% at 25 µg/mL total AVNs, which is illustrated in Figure 3A. It also showed a dose-dependent manner. The ROS production tends to be decreased (*p* < 0.05) in regard to the co-treatment of the GOEs (1 to 50 µg/mL) with Aβ_1-42_ for 24 h in the SH-SY5Y cells. It was particularly reduced to 155.85 ± 18.68% at 1 µg/mL, 131.39 ± 16.42% at 10 µg/mL, 108.36 ± 15.81% at 25 µg/mL, and 91.78 ± 15.89% at 50 µg/mL GOEs, which are depicted in Figure 3B. A remarkable elimination in the ROS induced by Aβ_1-42_ occurred with a co-treatment of 25 μg/mL AVNs and at 50 μg/mL GOEs. However, 1 and 10 µg/mL GOEs did not show any significant differences from only the Aβ_1-42_ treatment (*p* < 0.05).

A previous study revealed that the generation of ROS or free radical-induced oxidative stress can cause damage to cells and tissues, which is parallel with a loss in the functional efficiency of various cellular processes that results in aging and age-related memory loss [40]. According to the hypothesis about Aβ fibril, Aβ_1-42_ fibrils, which were entrapped by metal ions, such as copper ions, during the aggregation procedure, are considered toxic agents due to their contribution to the ROS generation that involves the oxidation of lipids, proteins, and nucleic acids [41]. ROS, which was in the mitochondria, leads to attacking the membrane phospholipids, and it contributed to the loss of the mitochondrial membrane by producing the intermembrane protein, such as cytochrome c triggers with activation of caspase-3 [42,43]. It was recently found that AVN C especially influenced the decrease in cleaved-caspase-3 activity in mouse hippocampus, which was reacted with the activated-caspase-3 that causes apoptotic cell death [37]. Similar results were found in a previous study, which evaluated the protective effects of AVN A and oat seeding extracts on the ROS production to lipopolysaccharide (LPS)-stimulated BV2 microglial cells, in which the levels of the ROS were significantly diminished [25]. The current study showed that the Aβ_1-42_-induced ROS generation was partly diminished by the AVNs and GOEs.

### 3.5. Effects of AVNs and GOEs Against Aβ_1-42_-Induced Inflammation and Apoptosis of SH-SY5Y Cell

**Figure 4:**
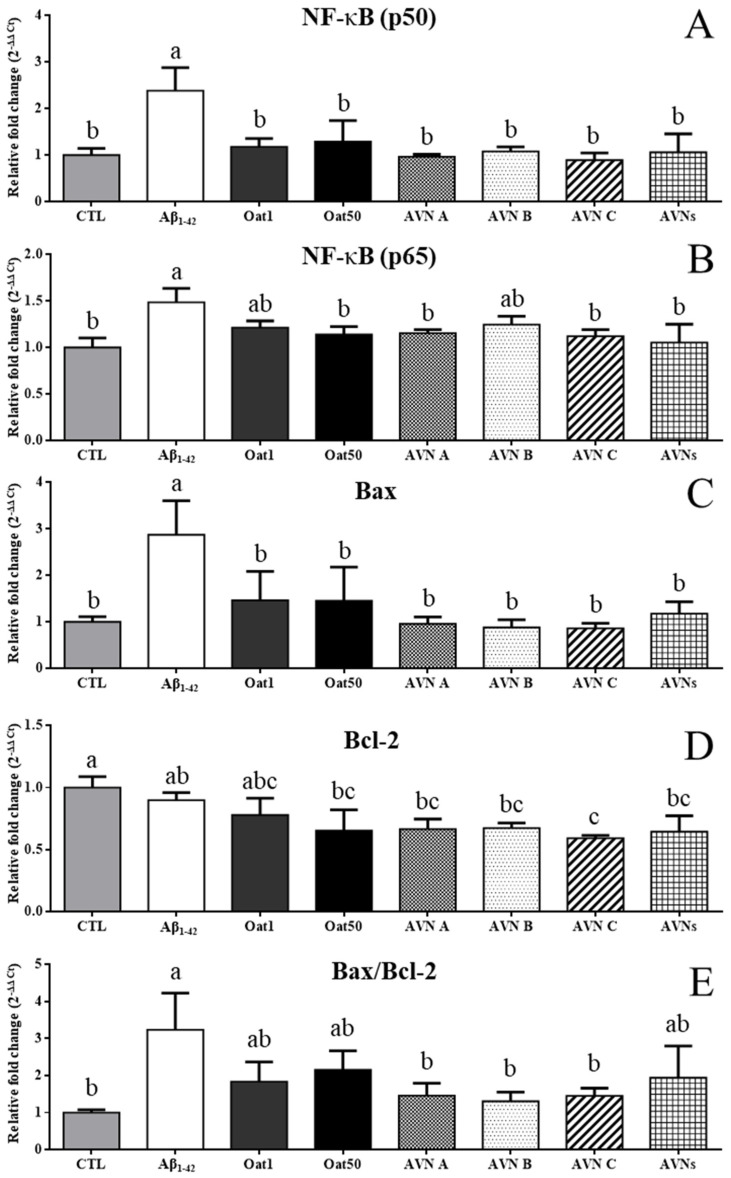
Expression levels of NF-κB, Bax, and Bcl-2 mRNA in SH-SY5Y cells with the sample treatment for 24 h, and Aβ_1-42_ (1 μM) was inoculated for a further 24 h. (**A**–**E**) The relative expression levels of NF-κB (p50), NF-kB (p65), Bax, Bcl-2, and Bax/Bcl-2 ratio. The data was presented as mean ± SD. The different letters (a, b, c) indicate significant differences among each treatment group (*p* < 0.05). Data are presented as mean ± standard deviation (SD) of [n = 3] independent experiments. Statistical significance among treatment groups was determined using one-way ANOVA (or two-way ANOVA where applicable), followed by Tukey’s honestly significant difference (HSD) post hoc test.

The current study evaluated the mRNA expression levels of NF-kB p50 and p65 in order to investigate biomarkers in regard to preventing the effect of AVNs and GOEs against Aβ_1-42_-induced neuroinflammation. The mRNA expression levels of NF-kB p50 were significantly increased to 2.38 ± 0.49 at Aβ_1-42_, as shown in Figure 4A. On the other hand, the AVN and GOEs treatment groups showed p50 mRNA levels of 1.17 ± 0.18 at OAT1, 1.29 ± 0.45 at OAT50, 0.97 ± 0.05 at AVN A, 1.08 ± 0.1 at AVN B, 0.89 ± 0.16 at AVN C, and 1.06 ± 0.4 at AVNs, which significantly recovered the expression levels (*p* < 0.05). The Aβ_1-42_ induced a higher p65 expression level of 1.49 ± 0.15, but OAT 1 and AVN B treatment groups tended to slightly decrease p65 mRNA levels of 1.21 ± 0.07 and 1.25 ± 0.09, respectively, which are illustrated in Figure 4B. The AVN and GOEs treatment groups showed p65 mRNA levels of 1.14 ± 0.09 at OAT50, 1.15 ± 0.04 at AVN A, 1.12 ± 0.07 at AVN C, and 1.05 ± 0.2 at AVNs, which revealed that the expression levels of these groups were significantly recovered (*p* < 0.05). The current study also investigated whether the expression of Bcl-2 and Bax was affected by AVN and GOEs. Aβ_1-42_ significantly increased the expression levels of Bax by 2.87 ± 0.73, as shown in Figure 4C. On the other hand, a significant decrease in the Bax levels of the AVN and GOEs treatment groups were observed, which showed 1.46 ± 0.62 at OAT1, 1.46 ± 0.72 at OAT50, 0.96 ± 0.15 at AVN A, 0.88 ± 0.16 at AVN B, 0.86 ± 0.11 at AVN C, and 1.18 ± 0.26 at AVNs compared to Aβ_1-42_, which are shown in Figure 4C. The Bcl-2 levels tended to decrease in all groups compared to CTL, 0.9 ± 0.06 at Aβ_1-42_, 0.78 ± 0.13 at OAT1, 0.65 ± 0.17 at OAT50, 0.67 ± 0.08 at AVN A, 0.67 ± 0.04 at AVN B, 0.59 ± 0.02 at AVN C, and 0.64 ± 0.13 at AVNs, which are displayed in Figure 4D. The ratio between Bax and Bcl-2 proteins determines the mitochondrial apoptosis pathway. The Bax/Bcl-2 ratio also significantly increased in Aβ_1-42_ compared to CTL, as shown in Figure 4E. The GOEs and AVNs tended to decrease compared to Aβ_1-42_ at 1.83 ± 0.54 at OAT1, 2.15 ± 0.52 at OAT50, and 1.94 ± 0.86 at AVNs. AVN A, AVN B, and AVN C significantly decreased compared to Aβ_1-42_ at 1.46 ± 0.34 at AVN A, 1.31 ± 0.25 at AVN B, and 1.46 ± 0.21 at AVN C.

Inflammation is important for the host to recover from damage when the inflammation response is broken, but it can cause tissue damage, which results in the development of inflammatory diseases [44]. Aβ accumulation causes neurotoxicity, which triggers the release of inflammatory mediators [45]. Activation of the NF-κB pathway stimulates the release of inflammatory cytokines, such as TNF-α, IL-6, and IL-1β by inflammatory mediators [46]. NF-κB is a crucial nuclear transcription factor in all cells in order to regulate the gene expression that encodes various immune and inflammatory response mediators [47]. Apoptosis is a natural process in the development, but abnormal apoptosis contributes to the progression of neurodegenerative conditions [48,49]. Defects of the mitochondria, which are involved in Aβ-induced neuronal apoptosis, increased the production of ROS that causes oxidative stress, which is one of the major causes of AD pathology [50,51]. Bax coordinates with Bcl-2 in order to activate the mitochondrial apoptotic pathway [52], so the current study measured its biomarkers. These results indicate that AVNs and GOEs prevent against Aβ-induced inflammation in SH-SY5Y. The previous studies reported that Bcl-2 family proteins inhibited ROS-induced apoptosis, whereas the overexpression of pro-apoptotic Bax increased the ROS production [53,54]. The results demonstrate that AVN and GOEs exhibit the neuroprotective effect by attenuating Aβ-induced mitochondrial-dependent apoptosis in SH-SY5Y.

## 4. Conclusions

In conclusion, the findings in the current study suggest that germinated oat extracts (GOEs) enriched in AVNs could inhibit Aβ_1-42_-induced cytotoxicity and oxidative stress in SH-SY5Y cells. The protective effects of GOEs against cytotoxicity were observed. The ROS production that was induced from Aβ_1-42_ was highly observed from 25 μg/mL AVNs and at 50 μg/mL GOEs, which implies that the GOEs that contain AVNs and phenolic acids, such as p-coumaric acid and ferulic acid, could be effective in regard to protecting the ROS generation in SH-SY5Y against Aβ_1-42_. Our results revealed that AVNs and GOEs mitigated the Aβ_1-42_-induced cell damage by rescuing inflammation and apoptotic pathways, which thereby increased the cell viability. However, additional biomarkers that were related to the apoptosis, including cytochrome C, caspase 9, and caspase 3, and inflammation-related cytokines, such as TNF- α IL-6, and IL-1β, need to be further investigated via an in vivo model in order to use GOEs for health functional ingredients for improving memory function.

## Figures and Tables

**Table 1:** Primer lists for RT-PCR.

Gene	Forward Sequence	Reverse Sequence
NF-κB (p50)	GCAGCACTACTTCTTGACCACC	TCTGCTCCTGAGCATTGACGTC
NF-κB (p65)	TGAACCGAAACTCTGGCAGCTG	CATCAGCTTGCGAAAAGGAGCC
BCL-2	ATCGCCCTGTGGATGACTGAGT	GCCAGGAGAAATCAAACAGAGGC
BAX	TCAGGATGCGTCCACCAAGAAG	TGTGTCCACGGCGGCAATCATC
GAPDH	GTCTCCTCTGACTTCAACAGCG	ACCACCCTGTTGCTGTAGCCAA

**Table 2:** Analytical methods validation of AVN A, B, and C.

Parameters	AVN A	AVN B	AVN C
Linearity			
Concentration range (µg/mL)	1–50	1–50	1–50
Regression Equation	y = 55608x − 22077	y = 45200x − 14845	y = 35929x − 19394
Correlation coefficient, *r*^2^	0.9997	0.9998	1
Accuracy and Precision			
Accuracy (%)	98.82–100.47	100.17–102.70	99.70–101.59
Precision (CV%)	1.40–4.51	2.65–4.85	0.91–2.30
Sensitivity			
Limit of detection (LOD, µg/mL)	0.733	0.616	0.610
Limit of quantification (LOQ, µg/mL)	2.223	1.866	1.848

## Data Availability

Data is contained within the article.
